# Atrial fibrillation and metabolic syndrome: an updated review of mechanisms, risk factors, and therapeutic strategies

**DOI:** 10.3389/fcvm.2026.1666063

**Published:** 2026-05-19

**Authors:** Huiming Zou, Shan Xiao, Tian Zheng, Yanping Zhang, Xiaochun Song, Wei Gu, Biming Zhan, Qianghui Huang

**Affiliations:** 1 Department of Cardiovascular Medicine, Jiangxi Medicine College, The Second Affiliated Hospital, Nanchang University, Nanchang, China; 2 Department of Radiology, The Second Affiliated Hospital, Jiangxi Medicine College, Nanchang University, Nanchang, China; 3 Department of Respiratory Medicine, Jiangxi Medicine College, The First Affiliated Hospital, Nanchang University, Nanchang, China; 4 Guizhou Medical University, Guiyang, Guizhou, China

**Keywords:** atrial fibrillation, dyslipidemia, hypertension, insulin resistance, metabolic syndrome, obesity

## Abstract

In recent years, atrial fibrillation (AF) has emerged as one of the most prevalent cardiac arrhythmias with escalating global incidence. Characterized by complex pathophysiology and unfavorable clinical outcomes, AF has become a focal point in cardiovascular research. Concurrently, metabolic syndrome (MetS)—a clinical constellation encompassing obesity, insulin resistance or impaired glucose metabolism, hypertension, and dyslipidemia—has evolved into a major global public health challenge. Accumulating clinical evidence indicates that MetS confers a 1.5–2 times increased risk of AF development compared with the general population, underscoring intricate pathophysiological interconnections between these conditions. Although conventional perspectives attribute this association primarily to obesity and hypertension, emerging research implicates multifactorial mechanisms including myocardial fibrosis, atrial electrical-structural remodeling, systemic inflammation, oxidative stress, adipokine dysregulation, and autonomic nervous system dysfunction. Nevertheless, the association of MetS with AF remains incompletely understood, with ongoing debates regarding the independent contributions and synergistic interactions of individual metabolic components in modifying atrial substrate. This review synthesizes current epidemiological evidence, explores the molecular mechanisms of the MetS and AF interfaces, and evaluates recent advances in clinical intervention strategies, with the aim of informing early preventive approaches and precision therapeutics.

## 1 Introduction

Atrial fibrillation (AF), a supraventricular tachyarrhythmia, is associated with major complications such as stroke, heart failure, and cognitive dysfunction, and contributes to substantial healthcare burden (1). Clinically, AF is classified into paroxysmal AF (self-terminating within 7 days), persistent AF (lasting more than 7 days or requiring cardioversion), and postoperative AF (occurring after surgery, particularly cardiac surgery), each of which involves partially distinct electrophysiological and pathophysiological mechanisms (2).

Metabolic syndrome (MetS), a cluster of interrelated metabolic abnormalities, has risen in parallel with AF and shows a close epidemiological association with AF development (3, 4). According to the international standard consensus, MetS is diagnosed when at least three of the following indicators are present: central obesity, elevated blood pressure, elevated fasting glucose, elevated triglycerides, and reduced high-density lipoprotein cholesterol levels (5). Current evidence suggests that patients with MetS exhibit an increased risk of atrial electrical and structural remodeling. Furthermore, their associated inflammatory state, oxidative stress, dysregulated glucose and lipid metabolism, and autonomic nervous dysfunction may exacerbate atrial substrate heterogeneity (6, 7). Clinical studies further indicate that MetS constitutes an independent risk factor for AF occurrence and may also reduce the long-term success rate of treatments like catheter ablation (8). The AF risk in MetS patients is amplified by the cumulative effect of metabolic abnormalities over time. Accordingly, early identification and management of metabolic abnormalities are important for AF prevention (9, 10). Importantly, most existing studies have focused on individual MetS components, whereas the role of MetS as a composite condition and its integrated impact across different AF phenotypes remain less clearly defined. This review synthesizes current evidence on the epidemiological links, pathophysiological mechanisms, and clinical intervention strategies linking MetS and AF, aiming to provide a theoretical basis for the early prevention and precise management of AF.

**Figure 1:**
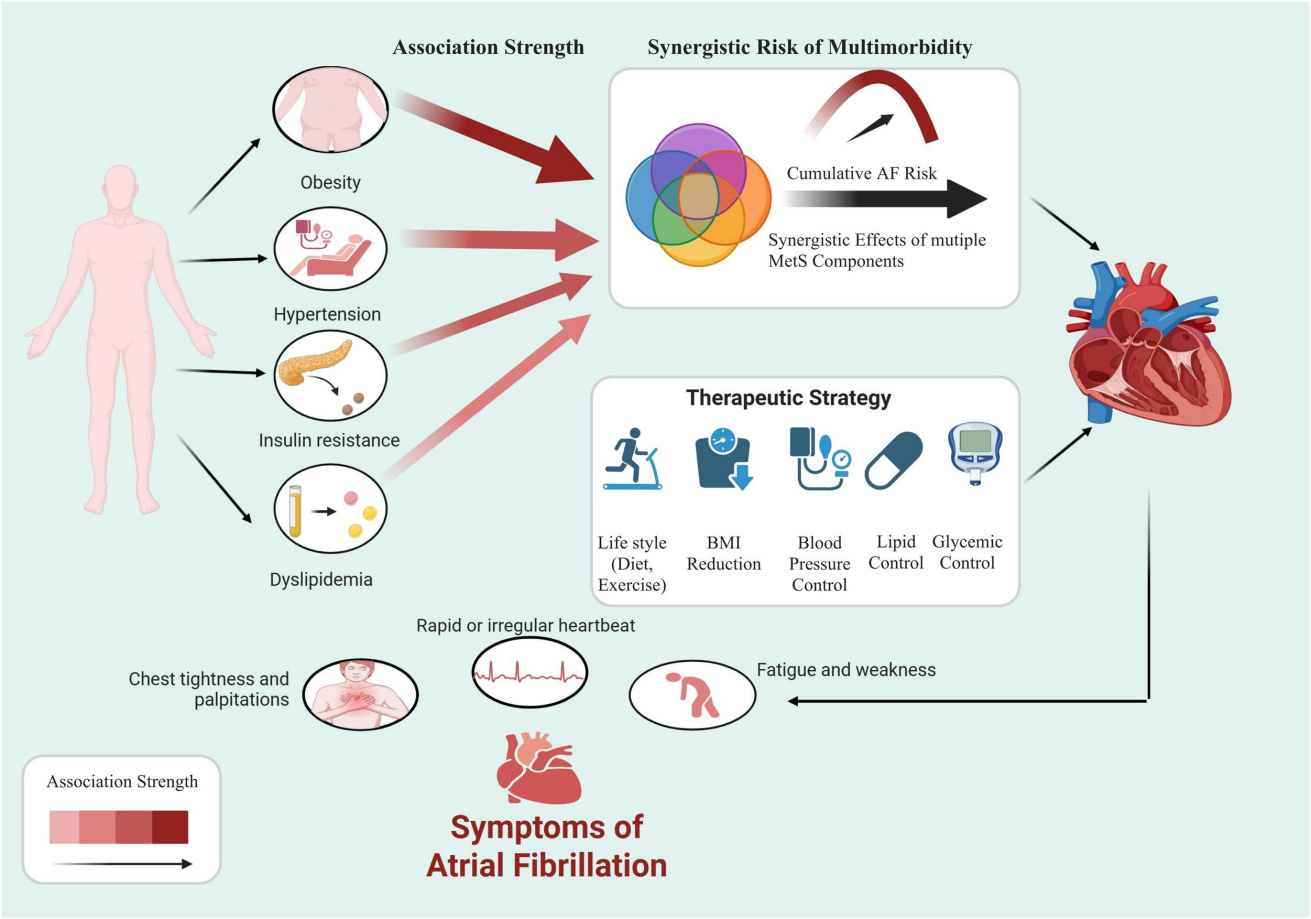
Schematic diagram of MetS leads to AF. (Created with BioRender.com).

Figure 1. Schematic diagram of MetS leads to AF.

## 2 Methods

### 2.1 Search strategy

This review followed the SANRA (Scale for the Assessment of Narrative Review Articles) guidelines for narrative reviews to ensure transparency and reproducibility (11). A narrative review was conducted to identify and summarize the available evidence on the link between MetS and its components to AF. The methodology was guided by best practices for literature-based reviews and emphasizes transparency of sources and synthesis. A comprehensive literature search was performed in PubMed, Web of Science, Cochrane Library and Embase databases, covering the period January 2000 to December 2025. Search terms included “atrial fibrillation”, “metabolic Syndrome”, “obesity”, “hypertension”, “insulin resistance”, “abnormal glucose metabolism”, “dyslipidemia”, “pathophysiology”, “mechanism”, “sex”, “Racial”, “Ethnic”, “Geographic Disparities”, “emerging therapies”, “treatment” and “radiofrequency ablation”.

Additional relevant publications were identified through manual searching of reference lists from key articles and narrative reviews. Two authors (HZ, QH) independently participated in all stages of screening. Disagreements were discussed with another author (SX) and resolved by consensus.

### 2.2 Study selection and eligibility criteria

This narrative review synthesizes and appraises the current understanding of the interplay between AF and MetS. Literature identification and selection employed a targeted approach guided by the following criteria: (1). Original research articles (clinical trials, observational studies, mechanistic studies), authoritative reviews, meta-analyses, and major clinical practice guidelines focusing on the epidemiology, pathophysiology, risk assessment, and therapeutic strategies linking AF and MetS; (2).While foundational literature provides context, emphasis is placed on evidence published within the last 10–15 years to capture advances in mechanisms, risk factors, and emerging treatments; (3).Studies examining the association between MetS as a whole and AF, as well as those exploring the contributions of individual MetS components (obesity, hypertension, dyslipidemia, insulin resistance) to AF pathogenesis and management.

Exclusion: (1) Articles exclusively focused on AF physiology without reference to MetS and its components; (2) case reports, editorials; (3) non-English publications.

Given the narrative nature of this review, formal systematic screening was not performed. Studies were evaluated for relevance based on the above criteria, and those making a substantive contribution to the topic were included. The key findings from major studies investigating the relationship between AF and MetS are summarized in Table 1.

**Table 1:** Summary of studies investigating MetS and AF.

Studies (Years of publication)	country	Study design	Population	Main results
Risk factors				
Thacher et al. (12)	Swedish, Danish, and Finnish	Cohort Study	161,115	The association for road traffic noise and AF appeared strongest in women and overweight and obese participants.
Yamanaka et al. (13)	Japan	Cohort Study	2,824	In patients with heart failure with HFpEF, the positive correlation between elevated BMI and AF risk is more pronounced.
Kim et al. (14)	Korean	Cohort Study	24,741	Hypertension in middle-aged East Asian men increases the risk of atrial fibrillation attacks.
Johnston et al. (15)	Canada	Cohort Study	771,521	Women exposed to HDP have a significantly increased cause-specific hazard ratios of incident AF.
Seyed Ahmadi et al. (16)	Swedish	Cohort Study	421,855	Individuals with type 2 diabetes had a higher risk of atrial fibrillation.
Ashburner et al. (17)	American	Cohort Study	2,101	Duration of diabetes is a more important predictor of ischemic stroke than glycemic control in patients who have diabetes and AF.
Lind et al. (18)	Swedish	Cohort Study	294,057	Fasting glucose at prediabetes levels is associated with development of AF.
Zhou et al. (19)	American	Cohort Study	5,365	Increased GV is associated with higher incidence of POAF
Chen et al. (20)	United Kingdom	Cohort Study	392,783	Low RC levels were associated with an increased risk of incident AF.
Ouyang et al. (21)	American	Cohort Study	15,792	Higher levels of RC are associated with an increased risk of AF.
Mohammadi-Shemirani et al. (22)	United Kingdom	Cohort Study	435,579	Lp(a) as a potential causal mediator in the development of AF.
Kaur et al. (23)	American	Cohort Study	6,238	Elevated lp(a) is associated with higher rates of cardiovascular outcomes.
Pol et al. (24)	American	Cohort Study	14,884	Higher levels of ApoA1 were independently associated with lower risk of ischemic cardiovascular outcomes.
Lee et al. (25)	Korean	Cohort Study	7,565,531	Dynamic changes in MetS status and persistent MetS were associated with an increased risk of AF in a large-scale Asian population.
Camm et al. (26)	United Kingdom	Cohort Study	477,904	The independent relevance of general adiposity for AF was more limited in men than in women.
Goergen et al. (27)	American	Cohort Study	441,047	The adjusted hazard of AF was similar between Black and White individuals with CIEDs.
Chamberlain et al. (28)	American	Cohort Study	15,094	MetS and most of its components were associated with a higher risk of AF.
Mechanisms				
Lubbers et al. (29)	Global/American	Review	–	In the context of obesity, AF is driven by the interrelated processes of inflammation, atrial remodeling, and oxidative stress.
Balan et al. (30)	Global	Review	–	Oxidative stress, inflammation, and mitochondrial dysfunction caused by obesity lead to atrial fibrillation.
Gao et al. (31)	Global (GWAS)	Mendelian randomization/Prospective	56,802	Leptin as an important mediator between BMI and AF.
Qin et al. (32)	United Kingdom	Cohort Study	478,479	New-onset cardiovascular events in both men and women was associated with pathophysiological pathways related to neutrophil degranulation and immunomodulation.
Wang et al. (33)	China	Cohort Study	86	Diabetes mellitus was associated with atrial structural remodeling, including atrium enlargement and atrial fibrosis
Arnò et al. (34)	China	Randomized Controlled Trial	192	Arrhythmias are caused by overactivation of the autonomic nervous system during metabolic stress in diabetics.
Andelova et al. (35)	Global	Review	–	Prevention or attenuation of oxidative and inflammatory stress can abolish the development of an arrhythmia substrate.
Ballestri et al. (36)	Global	Review	–	Pro-inflammatory, procoagulant, and pro-fibrotic mediators that may play an important role in the pathophysiology of cardiac and arrhythmia complications.
Itani et al. (37)	American	Cohorts study	75	Combination of both CMS and AF may be associated with a higher degree of inflammation than what is seen in either cardiometabolic syndrome or AF alone
López-Canoa et al. (38)	Spain	Cohorts study	322	The sex-based differences of FABP4 and leptin levels according to AF burden.
Therapeutics				
Noubiap et al. (39)	American	Cohorts study	1,415	Women with AF have a much greater benefit to total arrhythmia recurrence than men when managed with structured risk factors, and men are more inclined to AF reversal.
Rossello et al. (40)	Spain	Randomized Controlled Trial	6,874	In overweight or obese people with Mets, an ILI had no impact on the underlying structural and functional left atrial substrate measurements associated with AF risk.
Pop et al. (41)	Global	Review	–	Glucagon-like peptide-1 receptor agonists improve atrial electrical stability by modulating inflammatory and lipotoxic properties of EAT.
Fogari et al. (42)	Italy	Randomized Controlled Trial	391	Telmisartan was more effective than ramipril in reducing AF severity as well as in improving P-wave dispersion.

BMI, body mass index; HFpEF, preserved ejection fraction; GWAS, Genome-wide association studie; HDP, Hypertensive disorders of pregnancy; GV, glycemic variability; POAF, postoperative atrial fibrillation; RC, remnant cholesterol; Lp(a), Lipoprotein(a); ApoA1, Apolipoprotein A1; CIEDs, cardiac implantable electronic devices; CMS, cardiometabolic syndrome; ILI, lifestyle intervention; EAT, epicardial adipose tissue.

## 3 Epidemiological associations between MetS components and AF onset and their underlying pathological mechanisms

### 3.1 Obesity

Obesity has increased dramatically worldwide since 1975 and parallels the rising incidence of AF (29). As a central component of MetS, obesity is a well-established independent risk factor for AF, with each 1 kg/m^2^ increase in body mass index (BMI) associated with a 3%–8% higher risk of incident AF (43). Notably, even metabolically healthy obesity confers elevated AF risk (HR = 1.18), indicating that excess adiposity alone may contributes to arrhythmogenesis (44). Longitudinal data further demonstrate that adult weight gain independently increases AF risk (HR = 1.25) (45). Central obesity appears particularly important. Mendelian randomization studies show that visceral adipose tissue, rather than subcutaneous fat, is associated with incident AF (46). waist circumference (WC) defined abdominal obesity (WC ≥90 cm for men, WC ≥80 cm for women) increases AF risk independently of BMI (47). The “obesity paradox”, whereby obese AF patients exhibit lower short-term mortality, has been reported but remains unexplained (48, 49). Conversely, underweight status (BMI <18.5 kg/m^2^) is associated with increased recurrence after ablation, suggesting a U-shaped relationship between BMI and AF outcomes (50).

Mechanistically, obesity promotes AF through increased hemodynamic load, atrial enlargement, and chronic low-grade inflammation, leading to structural remodeling and heightened arrhythmogenic susceptibility (51–53).

Table 2 summarizes studies on the associations between Obesity, Waist Circumference, BMI, and AF occurrence.

**Table 2:** The impact of obesity, waist circumference, and BMI on AF occurrence.

Studies	Study design	Patients (n)	Population	Main findings
Kong et al. (54)	Retrospective Cohort Study	752,000	American GBD Study 2021 (1990–2021)	The global burden of high BMI-related AF varies across regions and time, threatening global health.
Lee et al. (44)	Retrospective Cohort Study	389,321	Korea (2004–2006) (mean age 45.6 years)	MHO individuals are at increased risk for AF development, and obesity was independently associated with elevated AF risk.
Munakata et al. (45)	Retrospective Cohort Study	16,444	Japan (2013–2022) (aged > 30)	Subsequent weight gain in adulthood has an interactive effect on new-onset AF.
Wang et al. (46)	Prospective Cohort Study	1,350,000	United Kingdom (2018–2021)	Individuals with a higher body fat percentage tend to exhibit a heightened genetic predisposition for susceptibility to AF.
Baek et al. (47)	Retrospective Cohort Study	501,690	Korea (2002–2013) (aged >18 years)	Abdominal obesity is an important, potentially modifiable risk factor for AF in nonobese Asian persons.
Ahn et al. (55)	Prospective Cohort Study	2,689	Korea (2018–2023) (median age 62 years)	Underweight patients showed a higher risk of AF recurrence after cryoablation compared with normal-weight patients.
Nteli et al. (48)	Retrospective Cohort Study	1,113	Greece (2015-2018) (aged >18 years)	BMI values outside the normal range were independently associated with poorer prognosis.
Marzak et al. (56)	Prospective Cohort Study	139	France (2017–2020)	Weight loss and management of risk factors are necessary to reverse the natural progression of AF to avoid EAT-mediated fibrotic irreversible remodeling.

BMI, body mass index; GBD, global burden of disease study; MHO, metabolically healthy obese; EAT, epicardial adipose tissue.

### 3.2 Hypertension

Hypertension is one of the most important and consistent risk factors for AF within MetS (57). Epidemiological studies show that hypertensive individuals have a 1.7–2.5-fold higher AF incidence than normotensive individuals (58). In a cohort of 24,741 East Asian men, hypertension increased incident AF risk by 43% (14). Long-term exposure to elevated blood pressure further amplifies risk, with systolic blood pressure ≥130 mmHg associated with a 56% increase in AF incidence (9). Blood pressure variability is also clinically relevant, as both excessively high and low levels are associated with adverse outcomes in AF patients (59). In women, hypertensive disorders of pregnancy (HDP) are associated with a 1.5 to 2-fold increased long-term AF risk (15). Moreover, the relationship between hypertension and AF is cumulative and time dependent. Chronic pressure overload leads to progressive atrial structural changes and increases susceptibility to arrhythmia (60).

Hypertension is associated with atrial fibrosis, chamber dilation, and electrical instability (61). Although AERP shortening is a hallmark of sustained AF, in some models of hypertension- or Ang II–induced atrial remodeling, AF susceptibility increases despite a prolonged AERP. These findings indicate that conduction slowing, fibrosis, and refractoriness heterogeneity are key drivers of AF incident in such contexts, as they promote the formation of re-entry circuits (62, 63).

### 3.3 Abnormal glucose metabolism/insulin resistance

Abnormal glucose metabolism and insulin resistance are strongly associated with AF risk. Large cohort studies demonstrate that type 2 diabetes mellitus (T2DM) increases AF incidence by approximately 35% compared with non-diabetic populations (16). Furthermore, the risk of AF exhibits a dose-dependent increase with longer duration of diabetes (17, 64). Lind et al. (18) demonstrated that even impaired fasting glucose and insulin resistance are associated with elevated AF risk, suggesting that early metabolic abnormalities contribute to arrhythmogenesis. Poor glycemic control further exacerbates this relationship. Patients with HbA1c > 7% exhibit higher AF risk and worse outcomes (17, 65). Glycemic variability (GV) has also emerged as an independent risk factor. A study by Zhou et al. (19) showed that patients with high GV demonstrate significantly increased risk of postoperative AF (POAF), with up to a 2.3-fold increase compared to those with stable glucose levels. GV is additionally associated with increased mortality in AF patients, independent of average glucose levels (66). Notably, younger women (<55 years old) with diabetes exhibit disproportionately higher relative AF risk, with a standardized incidence ratio (SIR) reaching 2.36 (16).

Studies show that T2DM is associated with structural changes, including increased left atrial volume and adverse remodeling, which may facilitate AF initiation and maintenance (67). At a pathophysiological level, hyperglycemia, insulin resistance, and metabolic stress promote inflammation, oxidative stress, and abnormalities in calcium handling, all of which contribute to electrical instability and atrial remodeling (68, 69).

### 3.4 Dyslipidemia

Dyslipidemia is a key manifestation of MetS, yet clinical studies suggested a complex, often paradoxical relationship with AF risk. An early study by Psaty et al. (70) found lower cholesterol levels were associated with an increased risk of developing AF, suggesting an AF etiology distinct from atherosclerotic pathways and introducing the “cholesterol paradox” concept (71, 72). Additionally, a large-scale prospective analysis (*n* = 392,783) demonstrated that lower remnant cholesterol (RC) levels were associated with an increased risk of incident AF (20). Collectively, these findings support an inverse association between circulating lipid levels and AF risk, suggesting that hypocholesterolemia may be linked to AF susceptibility. However, the above evidence is primarily derived from observational studies and therefore does not establish causality. Despite their scale and partial prospective design, residual confounding by unmeasured lifestyle factors or comorbidities like obesity limits causal inference (73).

Conversely, recent evidence suggests that certain lipid fractions may increase AF risk. Elevated RC and lipoprotein(a) [Lp(a)] levels are associated with increased AF incidence and adverse outcomes, including death and myocardial infarction (21–23). Different lipid components appear to exert heterogeneous effects, with VLDL potentially contributing to AF susceptibility, whereas ApoA1 may have protective effects (24, 71). Triglycerides, despite being a defining component of MetS, show only weak independent associations with AF (9).

Overall, dyslipidemia likely influences AF through indirect mechanisms, including systemic inflammation, oxidative stress, and metabolic dysregulation, rather than acting as a primary driver (74, 75).

## 4 Relationship between MetS as a clinical syndrome and AF

### 4.1 Mets and risk of incident AF

When the MetS is analyzed as a composite clinical entity (≥3 components), rather than as a collection of isolated risk factors, its association with AF becomes more evident (3, 14, 28). In a comprehensive meta-analysis of prospective cohort studies, individuals with MetS exhibited a 57% higher risk of incident AF compared with those without MetS, supporting the concept that the clustering of multiple metabolic abnormalities confers a synergistic rather than additive risk (9). Similarly, in the Atherosclerosis Risk in Communities (ARIC) cohort, MetS was associated with a higher incidence of AF, although the strength of this association appeared to vary by race (28). In a large Korean cohort of middle-aged East Asian men, MetS also predicted new-onset AF, further supporting the epidemiological relevance of MetS as a unified syndrome rather than merely the sum of obesity, hypertension, dysglycemia, and dyslipidemia (14). Consistent findings have also been reported in other Asian populations, including a nationwide Japanese registry study, where MetS remained an independent predictor of AF after adjustment for traditional cardiovascular risk factors, suggesting that the composite metabolic phenotype carries prognostic significance across diverse ethnic groups (76).

Importantly, the excess risk associated with composite MetS likely reflects the simultaneous amplification of atrial stretch, fibrosis, inflammation, oxidative stress, autonomic imbalance, and electrical heterogeneity, and MetS should be interpreted as a pathobiological milieu that promotes atrial vulnerability, rather than as a simple arithmetic addition of individual cardiometabolic traits (9, 30).

### 4.2 Mets and AF recurrence after rhythm control

In contrast to incident AF, recurrence occurs in patients with established disease and reflects the ability to maintain sinus rhythm after rhythm-control interventions (77). Importantly, recurrence is determined not only by systemic metabolic burden but also by pre-existing atrial substrate remodeling (78). An analysis including 12,924 patients showed that MetS was associated with a significantly higher risk of AF recurrence after catheter ablation (RR = 1.63) (79). Available studies indicate that AF phenotype influences outcomes. In cohorts with both paroxysmal and non-paroxysmal AF, patients with MetS had worse post-ablation results, especially in those with advanced atrial disease (79, 80). Additionally, MetS was associated with more extensive atrial remodeling, particularly in persistent AF, suggesting that metabolic dysfunction contributes to an advanced arrhythmogenic substrate beyond structural changes alone (78, 81).

Accordingly, paroxysmal AF, persistent AF, and post-ablation recurrent AF represent distinct but overlapping clinical entities along a continuum of atrial disease progression (82, 83). Although MetS adversely affects rhythm-control outcomes across this spectrum, the underlying mechanisms are likely heterogeneous: trigger-driven activity and autonomic imbalance may predominate in paroxysmal AF, whereas atrial fibrosis and electroanatomic remodeling play a more prominent role in persistent AF and recurrent disease after ablation (77, 78, 84).

### 4.3 Mets and postoperative AF

Postoperative atrial fibrillation (POAF) was considered distinct from both incident AF and post-ablation recurrence, as it develops within a unique perioperative milieu characterized by acute systemic inflammation, heightened adrenergic activation, oxidative stress, electrolyte imbalance, and rapid fluid shifts (85). These transient yet profound perturbations create a temporally defined window of atrial vulnerability, differing from the chronic substrate evolution observed in community-onset AF (86).

Although the evidence base is less extensive than that for incident AF in the general population, accumulating data indicate that MetS is clinically relevant in the perioperative setting. In a retrospective study of 756 patients undergoing coronary artery bypass grafting (CABG), MetS was identified as an independent predictor of POAF and was associated with prolonged hospitalization and increased postoperative complications (87). In addition, several studies have demonstrated that individual components of MetS, particularly obesity, insulin resistance, and hypertension, are associated with an increased risk of POAF following cardiac surgery (88, 89).

POAF can be interpreted within a substrate–trigger framework, in which a pre-existing vulnerable atrial substrate interacts with acute perioperative stressors (90). Surgical trauma induces a robust inflammatory response, with elevated circulating cytokines such as IL-6 and C-reactive protein (CRP), both of which have been linked to POAF incidence (91, 92). In this context, MetS may increase susceptibility to POAF by lowering the threshold for atrial instability before surgery (93).

### 4.4 Metabolic burden, disease progression, and clinical outcomes in AF

MetS contributes to both the development of AF and its adverse clinical outcomes through a complex interplay of metabolic, inflammatory, and prothrombotic mechanisms (53, 94). Compared with individual risk factors, the clustering of metabolic abnormalities creates a more pronounced pathophysiological milieu characterized by endothelial dysfunction, platelet activation, oxidative stress, and atrial fibrosis, thereby accelerating AF progression and increasing susceptibility to complications such as ischemic stroke, heart failure, and cardiovascular mortality (95, 96). For example, in AF populations with low CHA₂DS₂-VASc scores, the presence of MetS has been associated with a significantly increased risk of ischemic stroke (HR = 1.19), suggesting that metabolic burden confers additional risk beyond conventional stratification (97). Importantly, a dose response relationship has been consistently observed, whereby each additional MetS component increases the risk of incident AF by approximately 20%–30% (9). Furthermore, cumulative exposure to MetS over time is a critical determinant of disease trajectory. In a nationwide cohort study involving 2.88 million individuals, each additional year of MetS burden was associated with a 7% increase in AF risk, underscoring the importance of longitudinal metabolic stress rather than single time-point assessment (10).

Longitudinal analyses indicate persistent MetS confers the higher risk of AF occurrence and progression, while improvement in metabolic status reduces risk but does not fully normalize it, suggesting a “metabolic memory” effect (25).

Collectively, these findings support the concept that MetS should be regarded as a dynamic and cumulative exposure state that influences both AF onset and its long-term clinical outcomes.

## 5 Sex differences and population disparities in AF and MetS

Epidemiological studies indicate a higher AF prevalence in men; however, sex-specific differences in metabolic signaling and body composition play a critical role in modulating AF risk and outcomes in the context of MetS (98, 99). A UK Biobank study including 23,134 incident AF cases found that adiposity measures were more strongly associated with AF in women, whereas lean mass was more strongly associated in men, suggesting sex-dependent metabolic remodeling pathways (26). Furthermore, women exhibit higher circulating levels of fatty acid-binding protein 4 (FABP4) and leptin, which are linked to adipose-driven inflammation, atrial fibrosis, and electrophysiological remodeling, contributing to increased AF burden (38).

Sex disparities also extend to treatment. Women showed worse outcomes after catheter ablation in a multicenter randomized trial, possibly reflecting more advanced atrial remodeling and altered metabolic substrate utilization at intervention (100). Additionally, cardiorespiratory fitness confers greater protection against arrhythmia recurrence in females, whereas males show a trend toward higher AF reversal, suggesting sex-specific interactions among metabolic fitness, autonomic tone, and atrial substrate (39).

Compared with sex differences, racial/ethnic and geographic disparities are less directly linked to well-defined metabolic mechanisms. Black individuals have lower AF prevalence than White individuals despite higher rates of hypertension and obesity; this condition may relate to differences in body composition, autonomic regulation, genetic factors, and potential under-detection (27, 101). Australasia exhibits a higher age-standardized incidence rate (3.08 per 100,000 person-years), whereas South Asia demonstrates a comparatively steeper increase in incidence (annual increase of approximately 1.76%), which is higher than that observed in regions such as Western Europe (0.85%) and North America (0.92%), possibly due to lower BMI and lifestyle factors (54, 102–104).

MetS also shows marked population variation, with higher prevalence among Hispanic and certain Indigenous groups and a rising burden in non-Hispanic Asian populations (105). Globally, AF burden remains highest in high-income regions, while the fastest growth occurs in low- and middle-income countries, paralleling the increasing prevalence of MetS (106, 107). However, these patterns are predominantly epidemiological, with limited mechanistic insights into how metabolic signatures affect the risk of AF in specific populations.

## 6 Cellular and molecular mechanisms linking MetS to AF

### 6.1 Myocardial fibrosis and extracellular matrix remodeling

Myocardial fibrosis represents a central structural substrate linking MetS to AF (108). Fibrotic remodeling disrupts atrial architecture, increases conduction heterogeneity, and facilitates re-entry circuits (35). In MetS, multiple metabolic stressors, including obesity, hypertension, and insulin resistance, converge to promote fibroblast activation and extracellular matrix (ECM) deposition (30, 36). Transforming growth factor-β (TGF-β) signaling plays a key role in this process by stimulating fibroblast proliferation and collagen synthesis (109, 110). Experimental and clinical studies demonstrate that TGF-β1 expression is upregulated in diabetic and hypertensive states, contributing to atrial fibrosis and AF susceptibility (33, 109). Additionally, activation of the renin–angiotensin–aldosterone system (RAAS) enhances fibrogenesis through Ang II–mediated signaling pathways (111, 112). Ang II promotes fibroblast differentiation and increases collagen deposition via AT1 receptor activation (113).

Inflammatory mediators further amplify fibrotic remodeling. Cytokines such as IL-6 and tumor necrosis factor-α (TNF-α) stimulate matrix metalloproteinase (MMP) activity and ECM turnover, leading to structural disorganization (36, 110). Chronic low-grade inflammation, a hallmark of MetS, therefore acts synergistically with neurohormonal activation to accelerate fibrosis (37). Emerging evidence highlights the role of epicardial adipose tissue (EAT) in fibrosis. EAT secretes pro-fibrotic adipokines and inflammatory mediators that directly affect adjacent atrial myocardium (114, 115). This paracrine interaction promotes localized fibrosis and electrical uncoupling, further increasing AF susceptibility (116).

Taken together, fibrosis in MetS is not a passive process but reflects dynamic interactions among metabolic, inflammatory, and neurohormonal pathways.

### 6.2 Atrial electrical and structural remodeling

Atrial electrical and structural remodeling is fundamental to AF initiation and maintenance (117). In MetS, remodeling results from the combined effects of metabolic stress, fibrosis, and ion-channel dysregulation (30). Structural remodeling includes atrial enlargement, myocyte hypertrophy, and interstitial fibrosis, all of which contribute to conduction abnormalities (33). Electrical remodeling involves alterations in ion channel expression and function, and changes in calcium handling are particularly important (118). Diabetes is associated with downregulation of sarcoplasmic reticulum calcium ATPase 2a (SERCA2a), which impairs calcium reuptake into the sarcoplasmic reticulum, leading to intracellular calcium overload, prolonged action potential duration, and an increased risk of triggered activity (34, 119). This promotes delayed afterdepolarization and increases arrhythmogenicity (35). In addition, sodium and potassium channel remodeling contributes to conduction slowing and repolarization heterogeneity (117). Gap junction remodeling also plays a critical role. Long-term high-fat diet (HFD) induces atrial fat deposition and interstitial fibrosis, accompanied by downregulation of connexin 43 (Cx43) expression and may contribute to abnormal cardiac electrical conduction (120). These changes facilitate re-entry circuits, which are essential for AF maintenance (117). Structural and electrical remodeling are closely interrelated (121). Fibrosis creates conduction barriers, while ion channel dysfunction further increases dispersion of refractoriness (122).

Thus, atrial remodeling represents a key mechanistic bridge linking MetS to AF and reflects an integrated process involving structural, electrophysiological, and metabolic alterations.

### 6.3 Systemic inflammation

Elevated levels of inflammatory biomarkers, including IL-6, TNF-α, and CRP, are consistently observed in both MetS and AF populations (36). These mediators promote atrial structural remodeling by activating fibroblasts and enhancing extracellular matrix depositions, while also altering electrophysiological properties, thereby increasing arrhythmia susceptibility (75, 123, 124). Inflammatory signaling pathways such as nuclear factor kappa B (NF-κB) are activated in MetS, leading to transcription of multiple pro-inflammatory genes (35). NF-κB activation further enhances cytokine production and promotes oxidative stress, creating a self-amplifying inflammatory loop that sustains tissue injury (125). This chronic inflammatory environment accelerates atrial fibrosis, disrupts gap junction integrity, and contributes to electrical heterogeneity (30). Clinical evidence supports the mechanistic role of inflammation in AF. Elevated inflammatory markers are associated with both incident AF and recurrence following catheter ablation (126). In postoperative settings, acute inflammatory responses further increase AF risk, highlighting the interaction between systemic inflammation and transient physiological stress (85). Adipose tissue, particularly EAT, serves as an important source of inflammatory mediators (114, 115). This local inflammatory milieu promotes fibrosis and alters atrial electrophysiological properties (116).

In total, systemic inflammation acts as a important driver of atrial remodeling and arrhythmogenesis in MetS and represents a potential therapeutic target for AF prevention and management.

### 6.4 Oxidative stress and mitochondrial dysfunction

Oxidative stress plays a pivotal role in the development of AF in MetS (30). Increased production of reactive oxygen species (ROS) results from metabolic abnormalities such as obesity, hyperglycemia, and dyslipidemia (127). These conditions enhance activity of enzymatic ROS sources, including NADPH oxidase and uncoupled nitric oxide synthase, thereby amplifying oxidative burden in atrial tissue (128). Excess ROS disrupts cellular homeostasis, damages proteins and lipids, and promotes atrial structural remodeling (129). Mitochondrial dysfunction represents a major intracellular source of ROS in cardiomyocytes (130). Impaired mitochondrial oxidative phosphorylation leads to electron leakage from the electron transport chain, generating superoxide and other reactive species (131). Mitochondrial ROS contributes to calcium-handling abnormalities by modifying key calcium-regulatory proteins, thereby promoting intracellular Ca^2+^ overload and triggered activity (35).

In addition, ROS amplifies downstream pro-arrhythmic signaling, contributing to inflammation and fibrosis (69, 110). Oxidative stress also directly impairs ion channel function. Oxidative modification of L-type calcium channels and potassium channels alter depolarization and repolarization dynamics, increasing electrical instability (30, 132). Furthermore, lipid peroxidation products such as malondialdehyde (MDA) modify ion channel proteins and increase dispersion of repolarization, thereby facilitating early afterdepolarization and re-entry circuits (35, 74).

Overall, oxidative stress simultaneously affects structural, electrical, and metabolic pathways, acting as an integrative mechanism in AF pathogenesis.

### 6.5 Adipokine dysregulation and epicardial fat crosstalk

Adipose tissue functions as an active endocrine organ that secretes bioactive molecules, including leptin, adiponectin, and resistin, which influence cardiac structure and electrophysiology (133). In MetS, expansion of visceral adipose tissue alters adipokine secretion profiles, leading to a pro-inflammatory and pro-fibrotic milieu (134). Leptin levels are elevated in obesity and have been shown to promote inflammation and myocardial fibrosis through activation of JAK2/STAT3 and TGF-β signaling pathways (133, 134). Leptin also enhances sympathetic nervous system activity, further contributing to electrical instability (133). In contrast, adiponectin exerts anti-inflammatory and anti-fibrotic effects by inhibiting NF-κB signaling and reducing oxidative stress; therefore, reduced adiponectin levels in MetS are associated with increased AF susceptibility (135, 136). The imbalance between these adipokines contributes significantly to atrial structural remodeling (133). EAT plays a unique role due to its direct anatomical proximity to the atrial myocardium (114). In addition to local inflammatory signaling, it contributes to fibrosis and conduction heterogeneity through adipokine and lipid-mediated crosstalk (115). It secretes pro-inflammatory cytokines, chemokines, and free fatty acids that directly infiltrate adjacent atrial tissue, promoting fibrosis, inflammation, and conduction heterogeneity (137).

Recent studies further highlight the role of lipid accumulation and lipotoxicity in cardiomyocytes (138). Excess free fatty acids induce mitochondrial dysfunction, increase ROS production, and impair energy metabolism, thereby exacerbating atrial remodeling (75, 139). In addition, adipokine-mediated signaling interacts with oxidative stress and inflammatory pathways, amplifying arrhythmogenic substrate formation (30). Therefore, adipose tissue should be regarded not merely as an energy storage organ but as an important regulator of atrial pathophysiology.

### 6.6 Autonomic nervous system dysfunction

Autonomic nervous system (ANS) imbalance is a key contributor to AF in MetS (140). Metabolic disorders are characterized by increased sympathetic activity and reduced parasympathetic tone, resulting in a shift toward adrenergic dominance (141). This imbalance promotes atrial ectopic activity and facilitates the initiation of AF (142). Sympathetic activation enhances calcium influx through β-adrenergic signaling, increasing intracellular Ca^2+^ loading and promoting delayed afterdepolarizations and triggered activity (143). It also shortens the atrial effective refractory period and increases dispersion of refractoriness, thereby facilitating re-entry circuits (144). Conversely, reduced parasympathetic activity diminishes vagal-mediated stabilization of atrial electrophysiology, removing an important anti-arrhythmic influence (145).

Metabolic stressors such as hyperglycemia, insulin resistance, and obesity further exacerbate ANS dysfunction. Hyperinsulinemia stimulates central sympathetic outflow, while adipokines such as leptin directly activate sympathetic pathways (34). In addition, obstructive sleep apnea (OSA), which is highly prevalent in MetS, induces intermittent hypoxia and cyclic sympathetic activation, further increasing AF susceptibility (8, 146).

Experimental studies demonstrate that modulation of autonomic tone significantly influences AF inducibility, supporting a causal role of ANS dysfunction in arrhythmogenesis (147). Clinical observations further show that interventions improving autonomic balance, such as weight loss, exercise training, and β-blocker therapy, are associated with reduced AF burden (148, 149). Importantly, autonomic imbalance does not act in isolation but interacts with structural and electrical remodeling (150). Sympathetic overactivity promotes oxidative stress and fibrosis, while altered vagal tone affects ion channel function, together forming a complex arrhythmogenic substrate (151). Thus, autonomic nervous system dysfunction represents a critical mechanistic bridge linking metabolic abnormalities to AF.

**Figure 2:**
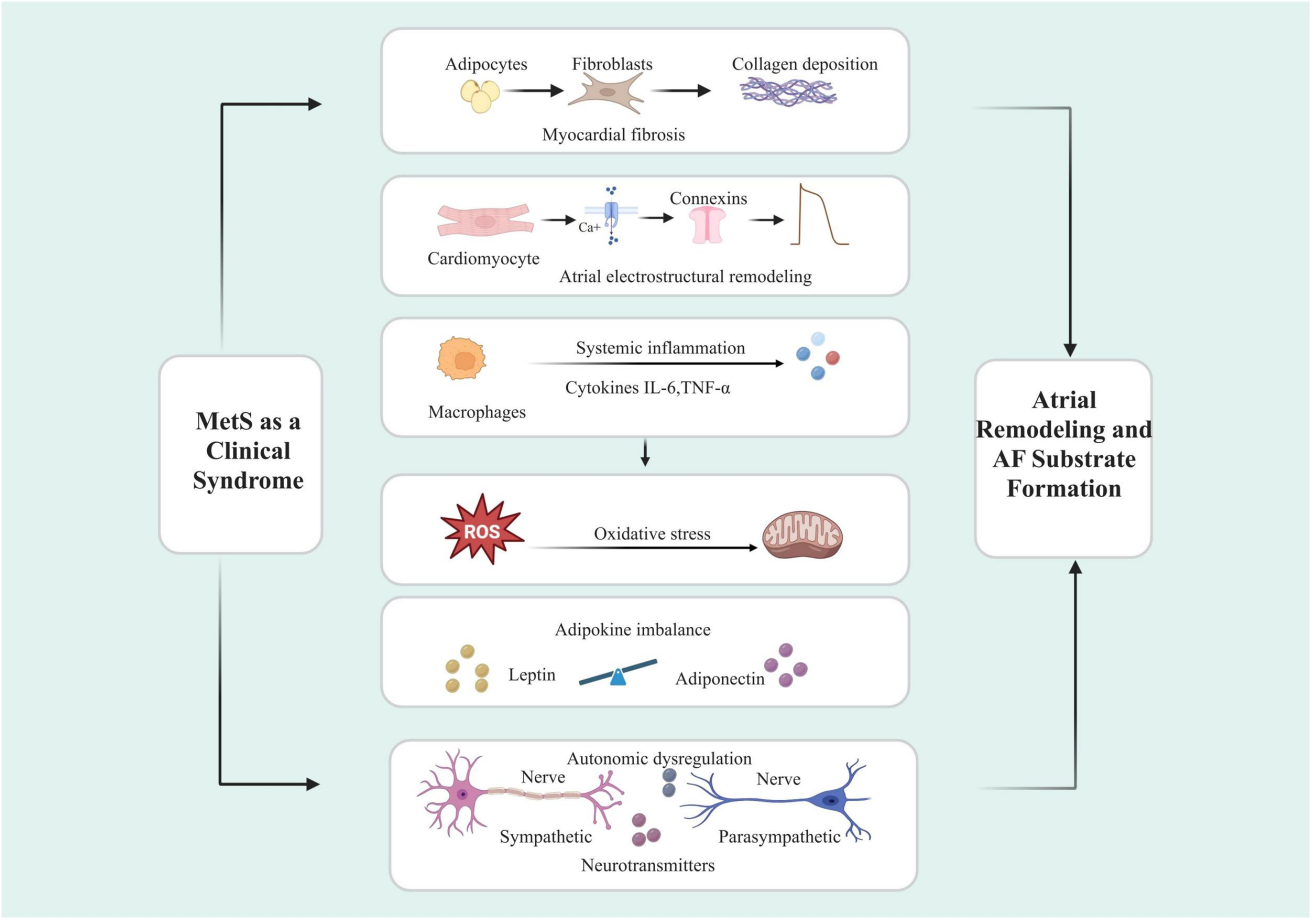
Molecular mechanisms linking MetS to AF. ROS, reactive oxygen species; IL-6, Interleukin-6; TNF-α, tumor necrosis factor-α. (Created with BioRender.com).

In summary, MetS represents a multifactorial pathophysiological milieu that facilitate AF through converging structural, electrophysiological, inflammatory, and metabolic disturbances, as conceptually illustrated in Figure 2.

## 7 Optimization of treatment strategies for metabolically related AF

### 7.1 Lifestyle-based risk factor modification underpins AF management in MetS

Lifestyle-based risk factor modification remains a cornerstone in the management of AF in patients with MetS (152). However, although weight reduction, structured exercise, and dietary optimization are consistently associated with lower AF burden and more favorable rhythm outcomes, current evidence does not uniformly support the view that intensive lifestyle intervention directly reverses atrial structural and functional remodeling in MetS populations (40, 153, 154). Rossello et al. (40) demonstrated that, in overweight or obese individuals with MetS, intensive lifestyle intervention did not significantly improve left atrial structural and functional parameters associated with AF risk, underscoring the need for cautious interpretation of its mechanistic effects. Therefore, lifestyle intervention should be regarded primarily as a strategy for global cardiometabolic risk reduction and AF burden control rather than as a universally effective approach for reversing atrial substrate abnormalities (155).

Nevertheless, sustained weight management remains one of the most clinically actionable upstream strategies, as weight loss has been consistently associated with reduced AF burden, improved maintenance of sinus rhythm, and enhanced outcomes following catheter ablation (152). Exercise training further contributes by reducing systemic inflammation, improving autonomic balance, and ameliorating insulin resistance, all of which are closely linked to AF pathogenesis (149, 156). Additionally, mediterranean-style dietary patterns may also improve the metabolic milieu, although their direct impact on atrial remodeling requires further validation (157). Collectively, these findings reinforce the role of lifestyle-based strategies as the therapeutic foundation of MetS-associated AF, while highlighting that their effects on atrial substrate are heterogeneous.

### 7.2 Comprehensive metabolic management of patients with AF

The success of catheter ablation in patients with MetS is strongly influenced by the underlying metabolic environment, as MetS is associated with higher rates of AF recurrence and more extensive atrial remodeling (57). Therefore, catheter ablation should be considered part of an integrated treatment strategy that includes comprehensive metabolic optimization rather than as a standalone intervention (81, 158). Importantly, effective management requires not only addressing individual metabolic abnormalities but also coordinating their treatment in a simultaneous and hierarchical manner (159).

Obesity and visceral adiposity should be prioritized as foundational targets because they exacerbate multiple downstream abnormalities, including insulin resistance, hypertension, and inflammation (160, 161). In parallel, glycemic control and blood pressure management are essential, as persistent metabolic dysregulation promotes ongoing atrial injury and fibrosis (73, 162). OSA is highly prevalent in MetS and represents an additional modifiable risk factor for AF recurrence, with studies demonstrating that untreated OSA significantly increases recurrence rates following ablation (8, 163, 164). Continuous positive airway pressure (CPAP) therapy has been associated with improved cardiovascular parameters and reduced AF recurrence, highlighting its importance in comprehensive management (165). Accordingly, a simultaneous yet hierarchical strategy integrating weight control, metabolic optimization, and OSA treatment is critical for improving ablation outcomes.

### 7.3 Metabolism-directed pharmacotherapy shows new directions

Pharmacological strategies targeting metabolic dysfunction have gained increasing attention as adjunctive therapies in AF management, particularly in patients with MetS (78, 97, 139). Among these, sodium–glucose co-transporter 2 inhibitors (SGLT2is) appear particularly promising due to their pleiotropic effects, including improved insulin sensitivity, reduced inflammation and oxidative stress, and decreased myocardial adiposity (78, 166, 167). Recent data suggested that SGLT2 inhibitor use is associated with a lower incidence of AF and may also reduce recurrence following catheter ablation in patients with diabetes (168, 169). Accordingly, these agents may be preferentially considered in MetS patients with coexisting diabetes, obesity, or heart failure (167).

Glucagon-like peptide-1 receptor agonists (GLP-1RAs) also represent a promising therapeutic class, particularly in obese or insulin-resistant individuals, as they promote weight loss, modulate EAT, and improve systemic metabolic profiles (170, 171). Emerging evidence suggests a potential reduction in AF risk with GLP-1RA therapy, although dedicated randomized trials focusing on AF-specific outcomes remain limited (172). In contrast, RAAS inhibition remains an upstream approach to consider, especially in patients with hypertension, where angiotensin receptor blockers may reduce atrial fibrosis and electrical remodeling (42). However, intensified or dual RAAS blockade remains controversial, and its additional benefit must be weighed against potential adverse effects such as hypotension and renal dysfunction (173, 174).

Additionally, adipokine-targeted therapies and gut microbiota–based interventions have emerged as potential strategies, but pharmacological approaches remain largely investigational and has not yet translated into routine clinical practice (51, 175, 176).

Overall, pharmacological strategies should be integrated within a broader metabolic framework rather than applied in isolation.

## 8 Conclusion

The growing public health burden of AF and MetS underscores the need for a clearer and clinically actionable understanding of their interaction. As outlined in this review, MetS is associated with an increased risk of AF across its clinical spectrum, including incident AF, recurrence after rhythm control, and postoperative AF, with risk progressively increasing alongside metabolic burden and its persistence over time.

Mechanistic evidence supports a multifactorial substrate involving myocardial fibrosis, atrial electrical and structural remodeling, systemic inflammation, oxidative stress, adipokine imbalance, and autonomic dysregulation. These interconnected pathways collectively promote atrial vulnerability, although their relative contributions likely vary across AF phenotypes. Accordingly, risk factor modification remains central. Weight reduction, blood pressure control, and glycemic optimization represent the most consistently supported strategies, complemented by management of comorbidities such as obstructive sleep apnea. Emerging evidence further supports a comprehensive metabolic approach integrating lifestyle, pharmacological, and interventional strategies, in which coordinated and prioritized targeting of metabolic abnormalities may be required to meaningfully alter AF trajectory. In addition, demographic variation appears to modulate the relationship between AF and MetS and warrant further investigation.

Future research should prioritize prospective, phenotype-oriented studies to clarify causal mechanisms and to define optimal integrated management strategies for MetS-associated AF.
